# Actions of a Proline Analogue, L-Thiazolidine-4-Carboxylic Acid (T4C), on *Trypanosoma cruzi*

**DOI:** 10.1371/journal.pone.0004534

**Published:** 2009-02-20

**Authors:** Anahí Magdaleno, Il-Young Ahn, Lisvane Silva Paes, Ariel M. Silber

**Affiliations:** Departamento de Parasitología, Instituto de Ciências Biomédicas, Universidade de São Paulo, São Paulo, Brazil; INSERM U567, Institut Cochin, France

## Abstract

It is well established that L-proline has several roles in the biology of trypanosomatids. In *Trypanosoma cruzi*, the etiological agent of Chagas' disease, this amino acid is involved in energy metabolism, differentiation processes and resistance to osmotic stress. In this study, we analyzed the effects of interfering with L-proline metabolism on the viability and on other aspects of the *T. cruzi* life cycle using the proline analogue L- thiazolidine-4-carboxylic acid (T4C). The growth of epimastigotes was evaluated using different concentrations of T4C in standard culture conditions and at high temperature or acidic pH. We also evaluated possible interactions of this analogue with stress conditions such as those produced by nutrient starvation and oxidative stress. T4C showed a dose-response effect on epimastigote growth (IC_50_ = 0.89±0.02 mM at 28°C), and the inhibitory effect of this analogue was synergistic (*p*<0.05) with temperature (0.54±0.01 mM at 37°C). T4C significantly diminished parasite survival (*p*<0.05) in combination with nutrient starvation and oxidative stress conditions. Pre-incubation of the parasites with L-proline resulted in a protective effect against oxidative stress, but this was not seen in the presence of the drug. Finally, the trypomastigote bursting from infected mammalian cells was evaluated and found to be inhibited by up to 56% when cells were treated with non-toxic concentrations of T4C (between 1 and 10 mM). All these data together suggest that T4C could be an interesting therapeutic drug if combined with others that affect, for example, oxidative stress. The data also support the participation of proline metabolism in the resistance to oxidative stress.

## Introduction

The protozoan parasite *Trypanosoma cruzi* is the causative agent of Chagas' disease, which affects approximately 12–14 million people in endemic areas throughout Mexico, Central, and South America [1]. The illness is characterized by an acute phase with patent parasitemia and non-specific symptoms (if any), followed by a life-long chronic phase with subpatent parasitemia and scarce tissue parasitism. In the symptomatic phase, the heart is primarily affected, developing hypertrophy and dilatation, in addition to the digestive tract – predominantly the esophagus and large intestine – with the appearance of megaviscera [2]. At present, the available therapy is mainly successful during the acute phase of the disease but with systemic side effects. Application of this therapy to treat the chronic phase of the disease – when most patients are diagnosed – is still controversial [3], 4.

*T. cruzi* has a complex life cycle characterized by several forms in vertebrate and invertebrate hosts [5], [6]. In the invertebrate host (the triatomine insect belonging to the family Reduviidae, order Hemiptera), the parasite replicates as a non-infective form called epimastigote, which can differentiate into a metacyclic trypomastigote, an infectious but non-replicative form. This process is called metacyclogenesis. When these forms infect a vertebrate host, they invade host cells and differentiate into amastigotes, which then divide in the cell cytoplasm and differentiate again into trypomastigotes, passing through a transient epimastigote-like stage termed the intracellular epimastigote [7], [8], [9]. The main transmission route of the parasite is via insect vector bites on any region of the host skin. During its feeding, the insect vector deposits feces and urine containing metacyclic forms, which are usually self-inoculated by the host when scraping the wound caused by the insect bite [10].

The successful survival of the parasite in different environments throughout its life cycle (e.g. the vector intestinal tract, the mammalian host blood stream and the host cell cytoplasm) depends on its ability to maintain intracellular homeostasis of ions and nutrients and the ability to catabolize different substrates to obtain energy. Thus, the activity of transporters allowing the uptake of solutes across the parasite cytoplasmic membrane and the metabolism of certain amino acids are crucial processes at various points in the *T. cruzi* life cycle [11], [4], [12]. Among these nutrients, several amino acids play important roles in many biological processes. Some metabolic pathways are particularly important under stress situations such as the interconversion between arginine and phosphoarginine by the enzyme arginine kinase [13]. This enzyme is regulated throughout the different growth phases [14] and is involved in resistance to pH and nutritional stress conditions [15] and in oxidative stress [16]. Other examples include the involvement of glycine, alanine, proline and glutamate in osmoregulation and cell volume control during all stages of the parasite [17], [18], in addition to the use of proline, glutamate and aspartate as energy and carbon sources [19] and in the metacyclogenesis process [20]. In particular, L-proline is an important metabolite that is involved in the differentiation of intracellular epimastigote to trypomastigote forms, which is required for the establishment of infection in the mammalian host [9].

Previous work showed that L-proline was taken up from the extracellular environment through two active transporters [21] and converted into five intermediates of the Krebs cycle (e.g., citrate, isocitrate, malate, succinate and oxaloacetate), pyruvate and the amino acids glutamate and aspartate, which are rapidly metabolized [19]. This seemed to comprise a conventional proline oxidation pathway. This idea is supported by the presence of two putative genes encoding proline oxidases and one putative gene encoding a pyrroline-5-carboxylate dehydrogenase annotated in the *T. cruzi* genome database [4]. Since L-proline participates in several differential processes in *T. cruzi* with respect to mammalian host cells, we hypothesize that inhibition of proline metabolism in *T. cruzi* could have more severe effects on the parasite biology that on host cells.

Various L-proline analogues such as T4C have been used as inhibitors of proline metabolism in prokaryote and eukaryote organisms [22], [23], [24]. In the present study, we analyzed the effects of T4C on the growth of epimastigotes and the infection of mammalian cells by *T. cruzi*. We also investigated the synergism or antagonism of T4C with stress conditions faced by *T. cruzi* during its natural life cycle: temperature, pH and oxidative and nutritional stress.

## Results and Discussion

### Growth inhibition assay

L-proline, a relevant energy source in trypanosomatids, is metabolized and converted into five intermediates of the tricarboxylic acid cycle (e.g., citrate, isocitrate, malate, succinate and oxaloacetate) through its oxidation to glutamate in *T. cruzi* [4]. Analogues of proline could be useful for inhibition of enzymes of this and other metabolic pathways. Among these analogues, L-thiazolidine-4-carboxylic acid, a product of the non-enzymatic condensation of equimolar quantities of formaldehyde and L-cysteine to yield the saturated imino acid containing S as a thioether [25], has the ability to interfere with the utilization of proline for protein synthesis and to mimic proline in its incorporation into proteins [26]. It can be metabolized in both bacterial and mammalian cells [26], [27], [28].

**Figure 1:**
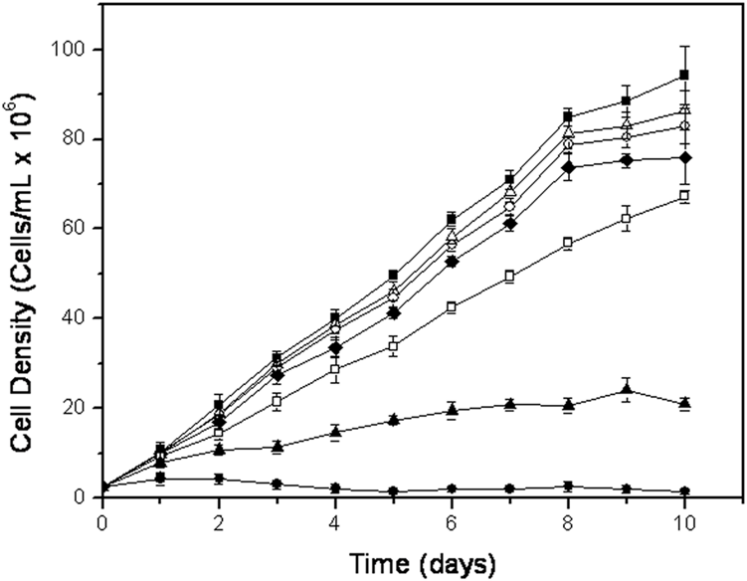
Growth curve of epimastigotes of *Trypanosoma cruzi* treated with T4C at 28°C and 7.5 pH: ▪ 0 mM, Δ 0.1 mM, ○ 0.25 mM, ♦ 0.5 mM, □ 0.75 mM, ▴1.0 mM. Inhibition control (•) was performed by incubation the parasites in the presence of 0.5 µM antimycin and 200 µM rotenone.

In order to investigate a possible trypanocide or trypanostatic effect of T4C on *T. cruzi*, 2.5×10^6^ epimastigotes per ml were cultured in the presence of different concentrations of T4C, ranging from 0.1 to 1 mM. The growth of epimastigotes on LIT medium with or without 200 µM rotenone and 0.5 µM antymicin were also followed as controls. The epimastigotes reached the stationary phase on the tenth day of growth, with 94×10^6^ cells/ml in LIT. T4C showed a dose-dependent inhibition of epimastigote growth at 28°C and pH 7.5, the standard temperature and pH for cultivating these cells. The observed growth differences between the treated cells and the LIT control were significant (*p*<0.01), and the IC_50_ was determined to be 0.89±0.02 mM (Figure 1), demonstrating an effect of T4C on *T. cruzi* epimastigotes.

It has been suggested that T4C inhibits the growth of *Escherichia coli* due to its specific interference with L-proline for protein synthesis by substituting L-proline in the corresponding prolyl-tRNA [27]. It has also been suggested that T4C could inhibit the synthesis of proline, thus enhancing the incorporation of this analogue into proteins [26]. In addition, when *Escherichia coli* is grown in a proline-free medium, the cells are able to actively oxidize T4C, and the product of this oxidation can be used as a carbon source for synthesis of the ribonucleic acid bases guanine and uracil [27]. In rat liver and kidney cells, T4C is oxidized by mitochondrial proline oxidase to L-thiazoline-4-carboxylic acid, which is then hydrolyzed to N-formyl-cysteine and later hydrolyzed to cysteine and formic acid by a cytosolic enzyme [28]. Likewise, T4C inhibited proline synthesis in the wilted leaves of plants that synthesize and accumulate proline rapidly during water stress conditions [29]. This inhibition had no effect on Δ^1^-pyrrolidine-5-carboxylic acid reductase (the enzyme that converts Δ^1^-pyrrolidine-5-carboxylic acid into proline). On the other hand, T4C had no influence on the incorporation of proline into proteins in plants. In addition, these authors found that concentrations between 0.2 and 1 mM T4C inhibited proline oxidation in mitochondria isolated from etiolated barley, and this inhibition was related to the activity of proline dehydrogenase and not the transport of proline into the mitochondrial matrix. All informations suggest that the inhibitory activity of T4C on *T. cruzi* growth could be due to any of the mentioned processes in which proline participates.

### Interaction with stress conditions

*T. cruzi*, *as well as* other parasitic organisms that cycle among different conditions and environments, must deal with radical physical, chemical and physicochemical variations such as temperature, availability of nutrients, oxidative stress and pH, all constituting stress factors. The interaction of T4C with these stress factors could identify a possible role for proline metabolism in *T. cruzi* biology, so we analyzed this interaction. As proline metabolism is involved in resistance to several stress conditions in a variety of organisms ranging from yeast to plants [30], [31], [32], we investigated if this amino acid could also be involved in resistance to such unevaluated stress conditions in *T. cruzi*. We initially evaluated the effects of T4C together with high temperature (33°C and 37°C), acidic pH (5.5 and 6.5), stress from nutrient deficiency (incubation in the presence of PBS supplemented or not with several carbon sources such as amino acids or glucose) and oxidative stress due to different reactive oxygen species (incubation in the presence of H_2_O_2_).

### 1) Temperature stress

**Figure 2:**
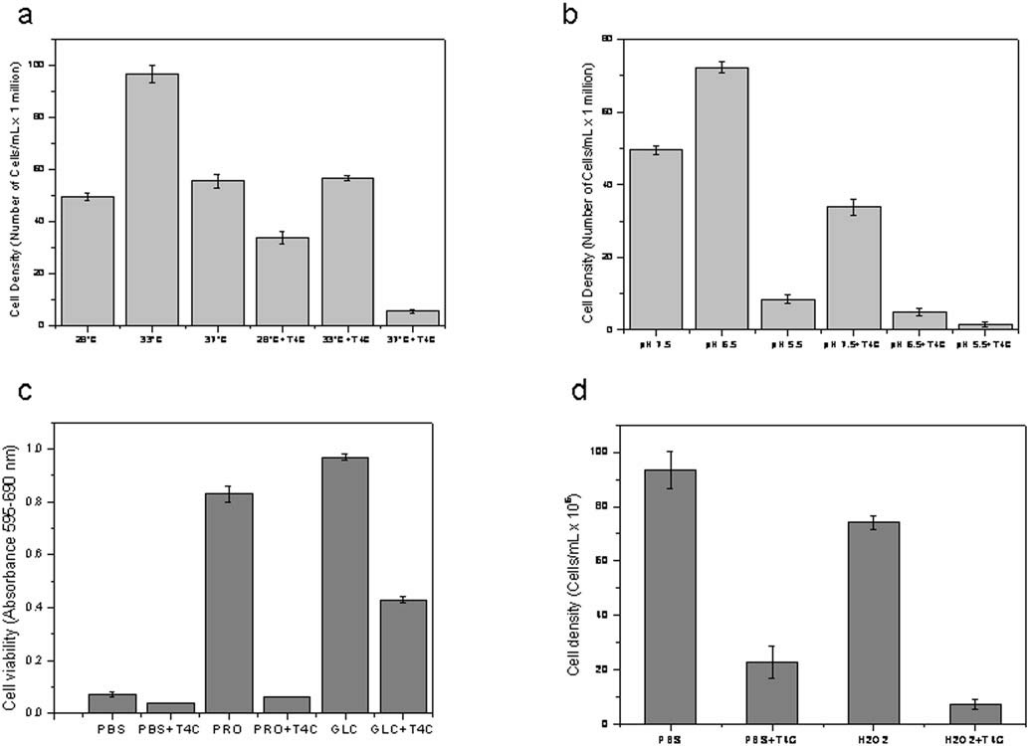
Response of epimastigotes at different stress conditions and T4C treatment. Panel a: the parasite density was measured at the 5^th^. day of growth, submitted or not to thermal stress (33°C and 37°C) and drug treatment (0.5 mM T4C). Panel b: the parasite density was measured at the 5^th^. day of growth, submitted or not to pH stress (pH 5.5 or 6.5) and drug treatment (0.5 mM T4C). Panel c) the parasite viability was measured by MTT assay after nutritional stresses performed by 72 h incubation in PBS, PRO and GLC, combined or not with the treatment with drug (0.5 mM T4C). Panel d: the parasites were submitted to oxidative stress by incubation in the presence of 160 µM H_2_O_2_ and the addition or not of drug (0.5 mM T4C). The effect of treatments and controls were measured by growing the parasites in LIT for five day and cell density, quantified.

As already mentioned, *T. cruzi* goes through temperature changes throughout its life cycle because the insect vector does not regulate its body temperature and because it alternates between an insect vector and a mammalian host. Therefore, this parasite faces environmental temperature variations and the high temperature of the mammalian body (up to 37°C). Epimastigotes grow optimally at a temperature of 28°C, a condition in which a cell density of 94×10^6^ cells/ml was reached at the stationary phase. Our data showed, however, that cell growth reached a higher density at 33°C (120×10^6^ cells/ml), while cell growth at 37°C was even less than that obtained at 28°C (70×10^6^ cells/ml). These results are in opposition to those obtained by [33], in which researchers reported an optimal temperature of epimastigote growth between 20°C and 28°C. The simultaneous effects of T4C and 33°C or 37°C diminished cell density in relation to that observed at 28°C (Figure 1). The IC_50_ values were calculated, being 0.83±0.07 mM and 0.54±0.01 mM for 33°C and 37°C, respectively (Figures S1, S2, S3, supplementary material). The simultaneous treatment with 0.5 mM of T4C and 37°C evidenced an effect for both variables that was significantly higher than the additive effects of T4C and temperature by themselves (Figure 2a), showing a synergistic interaction between these variables (*p*<0.05).

### 2) pH stress

*T. cruzi* also faces pH variations throughout its life cycle. In the intestinal tract of the insect vector, the pH varies between 5.9 and 8.9, depending on the starvation and blood meal periods [34]. About 2 months after feeding, the pH of triatomine excreta is acidic, and the parasite population in the feces is mostly constituted of metacyclic trypomastigotes. After feeding, the pH of excreta switches to alkaline, and one to five days later, it returns to a slightly acidic pH. During these periods, the population of epimastigotes increases, and the trypomastigotes and spheromastigotes forms are less common [35]. In addition, the metacyclic infective forms living in an acidic medium must face a slightly alkaline medium in the blood or inside the cell cytoplasm. Given this situation, it seemed interesting to evaluate how the parasites responded to acidic pH in the presence or absence of T4C. Epimastigotes were grown at pH 5.5, 6.5 and pH 7.5 as a control (i.e., the usual pH of LIT medium). It was found that their growth was strongly inhibited at pH 5.5, giving a maximum cell density of 21×10^6^ cells/ml in LIT medium, while parasites grown at pH 6.5 achieved similar cell densities (102×10^6^ cells/ml at the stationary phase) to those seen at pH 7.5 (94×10^6^ cells/ml) (Figure 2b). When the cultures were treated with T4C, the inhibitory effect on parasite growth was enhanced. It was observed that growth inhibition in the presence of T4C at pH 5.5 and 6.5 was stronger than at pH 7.5 (Figure 1). The IC_50_ values were calculated, being 0.58±0.03 mM at pH 5.5 and 0.51±0.03 mM at pH 6.5 (Figures S4, S5, S6, supplementary material). However, an interaction between the proline analogue and these growth conditions could not be confirmed, since the statistical test used to evaluate synergism was not significant (*p*>0.05). This result suggests that proline metabolism is not involved in pH stress resistance mechanisms.

### 3) Nutritional stress

Nutritional stress also occurs regularly during the *T. cruzi* life cycle, particularly in the gut terminal portion, where metacyclogenesis occurs [20]. It is well established that *T. cruzi* epimastigotes can metabolize not only proline, but also glucose, glutamate and aspartate. These energy sources are able to support the parasite survival to some extend after PBS starvation [19]. After nutritional stress, these metabolites are the fuel that energetically supports the parasite differentiation into the infective metacyclic forms [20], [36]. In order to analyze the possible effects of T4C on nutritionally stressed parasites, 20×10^6^ cells/mL in the exponential growth phase were starved for 72 h in PBS (PBS) or in 3 mM L-proline in PBS (PRO). Both cultures were treated or not (controls) with T4C, then cell viabilities were determined using the MTT assay. As expected, the presence of 3 mM proline in the PBS increased parasite viability when compared with parasites starved in PBS. Both cultures treated with T4C showed significant differences (*p*<0.01) compared to their respective controls (not treated with T4C), with a greater difference in the parasites maintained with L-proline (93%) compared to PBS (38%) (Figure 2c). This could be attributed due to either differences in the metabolic state of the cells or to a specific interference of T4C with proline metabolism. To clarify this point, a culture was also starved for 72 h with 3 mM glucose in PBS (GLC), then treated or not with T4C. As was observed (Figure 2c), treatment with T4C resulted in a 56% loss of viability, confirming that the effect of this analogue was higher in metabolically active parasites. The three situations of nutritional stress, however, showed a significant reduction in survival in the presence of T4C (*p*<0.01). Between these two essential nutrients incorporated in PBS, the most significant effect was obtained with glucose because it served as a carbon and energetic source. Other carbon and energy sources such as aspartate and glutamate could also have the same effect, since they seem to be as relevant as glucose or proline for the parasite survival in nutritional stress conditions [4], [19]. In spite of the various enzymes that are activated under nutritional stress conditions in order to generate free metabolites within the cell [37], [15], the viability of epimastigotes after 72 h was very low in PBS conditions.

### 4) Oxidative stress

In order to study the possible interaction between T4C and oxidative stress, we first evaluated the dose-response of the epimastigotes to different concentrations of H_2_O_2_. Cells (5×10^6^ parasites/mL) were resuspended in PBS and challenged with different hydrogen peroxide concentrations (between 0 and 200 µM) at 28°C for 90 min. After removal of the H_2_O_2_, cells were re-inoculated in fresh-LIT medium, and their growth was evaluated, as described in Materials and Methods. The concentration of H_2_O_2_ inhibiting 50% of parasite growth (IC_50_) was determined to be 160 µM under our experimental conditions. To determine the effects of H_2_O_2_ on T4C treatment, cells were then incubated under oxidative stress conditions (IC_50_) or in PBS in the presence or absence of 0.5 mM T4C, and parasite growth was followed (Figure 2d). The differences between each treatment (H_2_O_2_ or T4C) and its respective control were significant (*p*<0.01). The number of cells previously challenged with T4C was reduced by 55% with respect to the control, and cells incubated simultaneously in T4C and H_2_O_2_ showed a 75% reduction in growth. The protective effect of L-proline against oxidative stress was previously reported by some authors, such as [30] and [38]. Taking into account the effects of T4C on mammalian infection, it is possible that this could cause increased inhibition of the viability of infective forms. It was known that *T. cruzi* could generate different reactive oxygen species (ROS) by its own aerobic metabolism and the host immune response. These species lead to membrane disruption, inactivation of essential enzymes, mutagenesis and damage to the DNA repair machinery. The importance of these effects is primarily seen when the parasite invades the mammalian cell or when it is exposed to drugs used to treat Chagas' disease [39]. In the same way, T4C could act as a drug that increases the inhibitory effect of H_2_O_2_. In spite of the ROS detoxifying mechanisms in *T. cruzi* [40], [16], the epimastigote survival was low, at 0.5 mM T4C.

**Figure 3:**
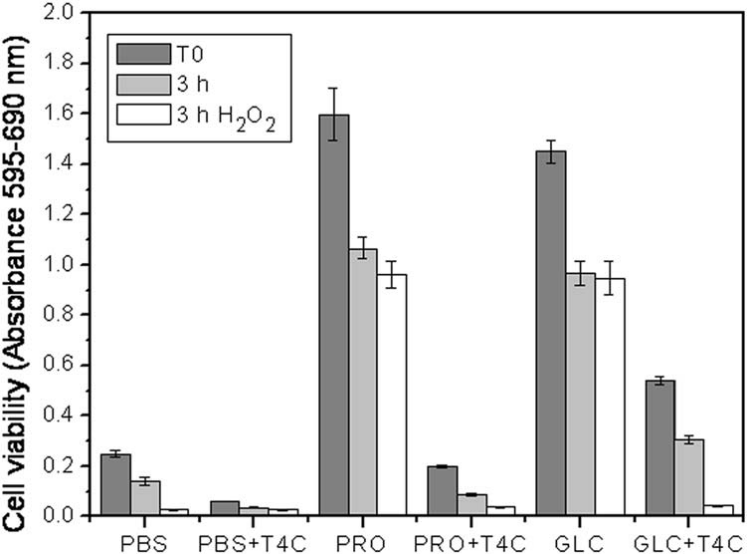
The effect of oxidative stress on epimastigotes previously incubated in PBS (PBS), L-proline 3 mM in PBS (PRO), and 3 mM Glucose in PBS (GLC), were treated or not with 0.5 mM T4C (PBS+T4C, PRO+T4C and GLC+T4C). After washing, the parasites were incubated for 3 h in PBS and stressed with 160 µM H_2_O_2_. The cells viability was evaluated by MTT assay.

From the results presented above, it could be proposed that the enhanced effect of T4C in the presence of H_2_O_2_ was due to the fact that this reagent affected a proline-dependent oxidative stress resistance mechanism. Alternatively, it could be argued that the enhancement of the effects of T4C seen under oxidative stress conditions was due to the fact that T4C made the cells more fragile by partially arresting proline metabolism, which is relevant for energy generation [19] and redox balance, as occurs in other organisms [30], [41], [38]. To better understand both the involvement of proline metabolism in resistance to oxidative and metabolic stresses and the interaction between these factors and T4C treatment, a three-variable experiment was performed: cell viability was evaluated as a function of oxidative and metabolic stress challenges and T4C treatment. Preliminary results forced us to diminish the metabolic stress by diminishing the starvation time from 72 (the time used above for metabolic stress experiments) to 48 h, since 72 h of starvation combined with oxidative stress and T4C treatment reduced viability to immeasurable levels. The parasites (20×10^6^ cells/mL) were incubated for 48 h in PBS, supplemented or not with 3 mM proline. As done previously, cultures maintained in PBS supplemented with 3 mM glucose were also evaluated. All groups were maintained in the presence or absence of 0.5 mM T4C (T4C), and the cultures were then challenged with H_2_O_2_, as previously described, or mock challenged with H_2_O. The cell viability in each case was evaluated by a MTT assay. Before the 3 h challenge (T_0_), the viability of parasites incubated with carbon sources (proline or glucose) differed significantly (*p*<0.01) from the viability of those incubated with PBS (Figure 3). As expected, T4C showed a synergistic effect with each nutrient condition: the cell viability was diminished by 75%, 88% and 62% in parasites starved with PBS, PRO and GLC, respectively. After a 3 h mock challenge, cell viability diminished between 33% and 55% with respect to T_0_, showing significant differences (*p*<0.01) in all of the treatments. When the T4C-treated parasites were challenged with H_2_O_2_, a significant reduction in viability was observed (*p*<0.01) compared to parasites that were mock challenged (Figure 3). Interestingly, the difference was not significant between the PRO or GLC groups. This pattern changed, however, when T4C was present, showing a reduction in cell viability after 3 h of incubation with H_2_O_2_ of 21% for PBS, 57% for PRO and 85% for GLC with respect to parasites not incubated under oxidative stress. Only simultaneous treatment with PRO-T4C and oxidative stress showed a significant difference with respect to PRO and oxidative stress (*p*<0.05).

**Figure 4:**
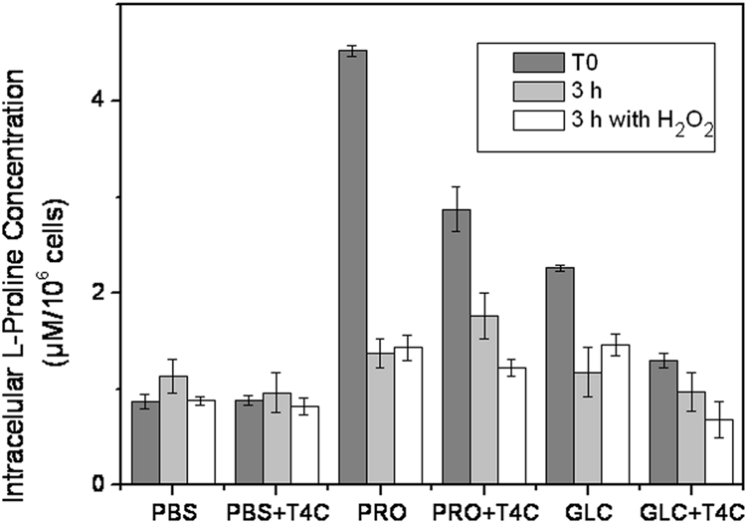
The intracellular proline concentration was measured as a function of nutrient starvation, oxidative stress and T4C treatment. The epimastigotes were incubated for 48 h in PBS (PBS), 3 mM L-proline (PRO) and 3 mM Glucose (GLC), supplemented or not with 0.5 mM T4C (PBS+T4C, PRO+T4C and GLC+T4C) (T_0_). After washings, the parasites were incubated along 3 h in PBS in the presence or not of 160 µM H_2_O_2_, and the intracellular concentration of proline was measured.

The enhanced effect of T4C on the PRO group with respect to the GLC group was expected and could be explained by the fact that T4C interfered with proline rather than glucose metabolism. In order to confirm this, we measured the intracellular free-proline levels in parasites starved for 48 h with PBS or 3 mM proline, in the presence or absence of T4C. Parasites starved with 3 mM glucose were used as controls, as previously described. After 48 h of incubation (T_0_), the intracellular free proline concentration was twice as high in the PRO group compared to the GLC group (4.52 and 2.26 µM/10^6^ cells, respectively) (Figure 4), indicating that when available, this metabolite could be transported and accumulated, as previously reported [21]. It is worth stressing that no differences were observed in cell viability between the PRO or GLC groups (p<0.05) (Figure 3). After 48 h of incubation (T_0_), the concentration of intracellular L-proline in the presence of T4C was maintained (PBS = 0.88 µM/10^6^ cells) or reduced (PRO = 2.87 µM/10^6^ cells, GLC = 1.29 µM/10^6^ cells) with respect to incubation without the analogue (Figure 4). The effect of the oxidative stress on the intracellular proline level was also evaluated by treating the cells with H_2_O_2_ (or H_2_O as mock stress) for 3 h. There were significant differences (p<0.01) in the intracellular L-proline concentration between the cells kept under oxidative stress and in the presence of T4C. These two results confirm the synergistic effects of T4C and oxidative stress. Higher sensitivity to H_2_O_2_ of the cells maintained in PRO/T4C with respect to those not treated with T4C could be attributed to lower intracellular free proline levels rather than to inhibition of its metabolism.

**Figure 5:**
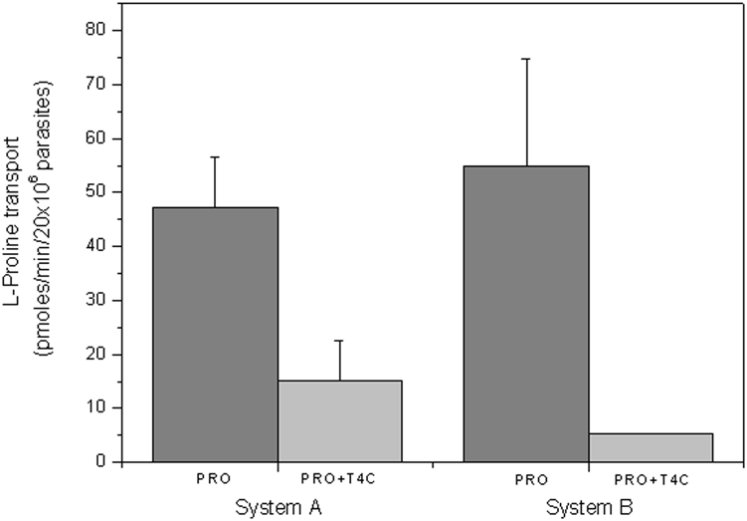
The effect of 0.5 mM T4C on L-proline uptake in epimastigotes of *T. cruzi*. Proline transport was measured at proline concentrations corresponding to the Km values for system A (0.31 mM) and system B (1.36 mM).

*T. cruzi* takes up proline through two active transport systems, one of them being a high affinity, low capacity transporter called system A, and the other a low affinity high capacity transporter called system B [21]. To further clarify the fact that L-proline is diminished by the presence of T4C, we performed a proline transport competition assay. Since T4C is a structural analogue of proline, the assay was designed under the hypothesis of a competitive inhibition, thus proline was used at concentrations in the range of the Km for each transport system, and T4C as a competitor was used at near its IC_50_ (0.5 mM) concentration for both assays. As can be observed, both transporters resulted partially inhibited (68% inhibition for system A and 91% inhibition for system B) (Figure 5). In turn, the proline dehydrogenase and proline racemase activities were not inhibited (data not shown).

### Effect of T4C on trypomastigotes bursting from infected host cells

**Figure 6:**
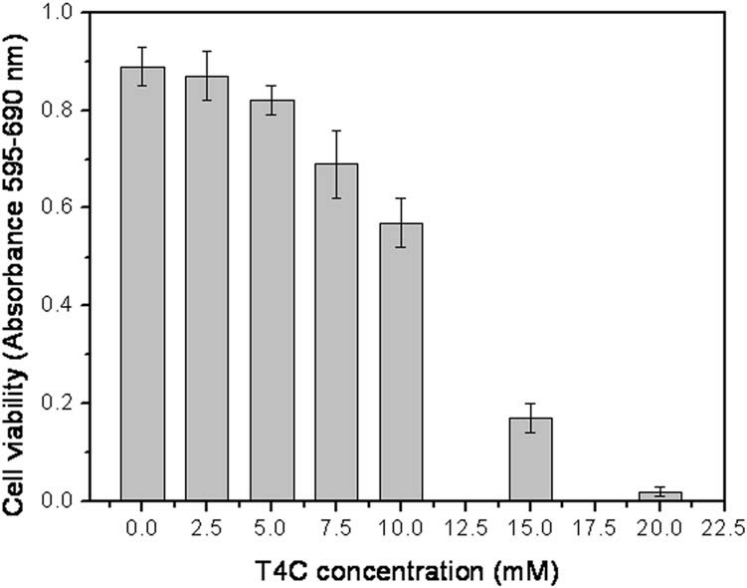
The effect of T4C on CHO-K_1_ line cells viability. CHO-K_1_ cells were challenged with different concentrations of T4C, and the cell viability was measured by MTT assay.

**Figure 7:**
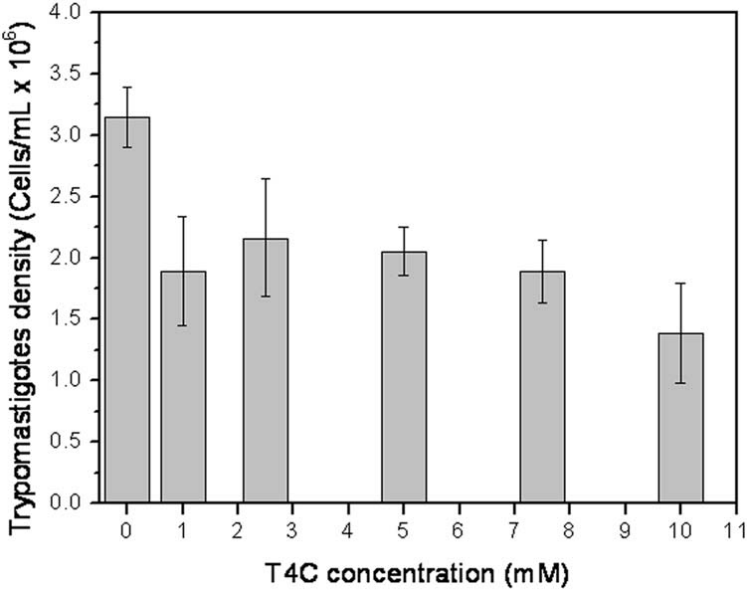
The effect of T4C on the trypomastigote bursting was evaluated. The trypomastigotes bursted at 5^th^. day post-infection in cell cultures treated or not with at different concentrations of T4C were counted.

In order to evaluate the effectiveness of treating the infected host-cells, the toxicity of T4C on mammalian CHO-K1 cells was determined. Interestingly, the inhibitory T4C concentrations tested on epimastigotes (between 0.1 and 1.0 mM) were not toxic to mammalian cells (IC_50_ = 11.4±0.36 mM) (Figure 6). It is worth mentioning that epimastigotes of *T. cruzi* are not infective stages, but they serve as a model to analyze whether similar effects would be seen in the infective trypomastigote stage. Therefore, the effects of the treatment of infected CHO-K1 cells were evaluated using concentrations up to 10 mM, causing a reduction of 31% and 56% in parasite bursting (Figure 7). The highest T4C concentration used to analyze survival through the intracellular stages of the parasite and the following release of trypomastigotes was 10 mM. The mammalian cells were then infected and treated with different concentrations of T4C (between 1.0 and 10.0 mM). After five days of infection, the number of trypomastigotes that burst into the extracellular control medium was approximately 3.1×10^6^ cells/ml. The parasite concentration in the presence of T4C was reduced to between 1.4×10^6^ cells/ml and 2.1×10^6^ cells/ml (Figure 7). There were significant differences between drug treatment and the control (*p*<0.05).

Proline is a relevant metabolite in *T. cruzi* biology, involved in several processes that are essential for the parasite life cycle [4]. Among them, differentiation in the insect vector stages [20] and inside the mammalian cell [9], as well as energy metabolism [19], seem to be of relevance. Taken together, these data are encouraging for the use of proline analogues able to inhibit the proline transport activity., on one side to identify new lead compounds for Chagas' disease therapy, and on the other side, to validate the metabolite transporters as drug targets [4], [12]. Further experiments are in process to analyze the synergic effects of T4C combined with nifurtimox and benznidazole, the drugs presently in use to treat this infection, with the aim of reducing their doses and perhaps diminishing their undesired side effects.

## Materials and Methods

### Reagents

L-proline, L-thyazolidine-4-carboxylic acid, rotenone, antimycin and MTT (3-(4,5-dimethylthiazol-2-yl)-2,5-diphenyltetrazolium bromide) were from Sigma (Saint Louis, MO, USA). L-[2, 3, 4, 5-^3^H] proline, 100 Ci/mmol was purchased from NEN Life Science Products (Boston, MA USA). RPMI culture medium and Fetal Calf Serum were from Cultilab (Campinas, SP, Brazil). All other reagents were from Amresco.

### Cells and parasites

The Chinese Hamster Ovary cell line CHO-K1 was routinely cultivated in RPMI medium supplemented with 10% heat-inactivated fetal calf serum (FCS), 0.15% (w/v) NaHCO_3_, 100 units mL^−1^ penicillin and 100 µg mL^−1^ streptomycin at 37°C in a humid atmosphere containing 5% CO_2_. Epimastigotes of *T. cruzi*, CL strain, clone 14 [42], were maintained in the exponential growth phase by subculturing every 48 h in LIT (liver infusion-tryptose) medium supplemented with 10% FCS at 28°C [43]. Trypomastigotes were obtained by infection of CHO-K1 cells with trypomastigotes, as described previously [7], [9]. Trypomastigotes were collected in the extracellular medium from five days onward.

### Growth inhibition assays

Epimastigotes in the exponential growth phase (approximately 50×10^6^ cells/mL) were washed three times by centrifugation and resuspended in phosphate buffered saline (PBS), then cultured in fresh-LIT medium supplemented or not (controls) with different concentrations of T4C ranging from 0.1 to 1.0 mM at 28°C. The assays were carried out in 96 well plates by inoculating 2.5×10^6^ cells/mL in 200 µL of medium. Cell growth was estimated by absorbance readings at 620 nm every day for ten days. The absorbance was transformed into a cell density value (cells/mL) using a linear calibration equation previously obtained under the same conditions (R^2^ = 0.9981, *p*<0.05). The concentration of T4C that inhibited 50% of parasite growth (IC_50_) was determined in the exponential growth phase (sixth day) by adjusting the effect (growth inhibition values) as a function of T4C concentration to a classical sigmoidal equation. As a cell growth inhibition control, growth curves in which 200 µM rotenone and 0.5 µM antimycin were added to the culture medium were run in parallel for all experiments.

### The effect of T4C on growth inhibition under stress conditions

To analyze the combined effect of T4C at different pH values and at different temperatures, the growth curves were generated as described above, with adjustment of the LIT pH to the desired values or by incubating the cultures at 28°C (no stress), 33°C and 37°C. To evaluate the effect of T4C combined with nutritional stress conditions, 20×10^6^ parasites/mL were washed and resuspended in 0.5 mM T4C in PBS (Phosphate Buffered Saline) or PBS supplemented with 3 mM glucose, 3 mM proline, or nothing (as control) for 72 h in Eppendorf tubes. Subsequently, cell viability was estimated by the MTT assay. To evaluate the combined effect of T4C and oxidative stress, 5×10^6^ parasites/mL in the stationary phase were maintained for 1 h and 30 min at 28°C in PBS or with 160 µM H_2_O_2_ in the presence or absence of 0.5 mM T4C. The cells were then collected by centrifugation and resuspended in LIT medium, and after 5 days, the number of cells/mL was determined as described previously [44]. A second experiment was carried out to evaluate the protective effects of L-proline and glucose (used as a control) on parasite viability under oxidative stress in the presence of T4C. In this experiment, 20×10^6^ epimastigotes/mL were washed twice, resuspended in PBS and pre-incubated for 48 h in PBS in the presence or absence of 3 mM L-proline and 3 mM glucose, with or without 0.5 mM T4C. After pre-incubation, the cells were washed twice, resuspended in PBS with or without 160 µM H_2_O_2_ and maintained under those conditions at 28°C for 3 h. Cell viability was then estimated using the MTT assay [45]. The L-proline concentration was measured before and after oxidative stress as previously described. To quantify free intracellular L-proline, the parasites were washed in cold PBS, suspended in 1 ml of PBS and lysed by sonication. The lysate was centrifuged (100,000 g, 30 min), and the supernatant was freed of proteins by trichloroacetic acid precipitation followed by centrifugation at 20,000 g for 30 min. Measurements of intracellular L-proline were made using the remaining supernatant solution, as described by Bates [46].

### Proline uptake and enzymatic activities

The proline competition assays were performed as previously described [47]. In brief: two-day cultured parasites were washed three times by centrifugation and resuspended in phosphate buffered saline (PBS), pH 7.4 at a final density of 200×10^6^ cells/ml and distributed in aliquots of 100 µl (containing 20×10^6^ cells each). Transport assays were initiated by the addition to the assay tubes of 100 µl of radioactively traced proline (0.5 µCi) to a final concentration of 0.31 mM or 1.36 mM, supplemented or not with 0.5 mM T4C. V_0_ was measured at 28°C for 30 s, and proline transport was stopped by addition of 800 ml of cold 50 mM proline in PBS, and rapid washing by centrifugation at 10,000 g for 60 s. The *T. cruzi* proline dehydrogenase activity was assayed as previously described [48]. Proline racemase was assayed in *T. cruzi* extracts by performing a coupled reaction, using D-proline as the enzyme substrate and L-proline dehydrogenase as the revelation system. Both assays were performed in the presence or absence of 0.5 mM T4C.

### Effect of T4C on mammalian cell viability

CHO-K1 cells (5.0×10^5^ cells ml^−1^) were inoculated in 24-well plates in FCS supplemented RPMI medium, as previously described, in the presence of increasing concentrations of T4C or no T4C (controls). The viability of cells was determined by the MTT assay, and the IC_50_ was obtained by fitting the data to a typical dose-response sigmoidal curve.

### Effect of T4C on trypomastigote bursting

CHO-K1 cells were grown on cover slips and infected with trypomastigotes in RPMI medium supplemented with 10% FCS. After 3 h at 37°C, free trypomastigotes in the medium were removed by washing with PBS, and the infected cells were maintained at 33°C in RPMI medium supplemented with 2% FCS, with or without different concentrations of T4C. These concentrations of T4C were not toxic to the mammalian cells. The trypomastigotes were collected in the extracellular medium on the fifth day and counted in a hemocytometer [9].

### Statistical analysis

A one-way ANOVA followed by Dunnet's test was used for statistical analysis. To analyze synergism between two independent treatments, a two-way ANOVA was performed as described previously [49]. A *p* value that was less than 0.05 was considered statistically significant.

## Supporting Information

Figure S1 Growth curve of epimastigotes of *Trypanosoma cruzi* treated with T4C at 33°C and 7.5 pH: ▪ 0 mM, ▵ 0.1 mM, ○ 0.25 mM, ⧫ 0.5 mM, □ 0.75 mM, ▴1.0 mM. Inhibition control (─) was performed by incubation the parasites in the presence of 0.5 µM antimycin and 200 µM rotenone.
(0.48 MB TIF)

Figure S2 Growth curve of epimastigotes of *Trypanosoma cruzi* treated with T4C at 37°C and 7.5 pH: ▪ 0 mM, ▵ 0.1 mM, ○ 0.25 mM, ⧫ 0.5 mM, □ 0.75 mM, ▴ 1.0 mM. Inhibition control (─) was performed by incubation the parasites in the presence of 0.5 µM antimycin and 200 µM rotenone.
(0.45 MB TIF)

Figure S3 Sigmoidal dose-response curve of epimastigotes of *Trypanosoma cruzi*. Percentage of growth inhibition of *T. cruzi* epimastigotes at different temperatures and T4C. The curves represent the sigmoidal equation at 28°C (—), 33°C (---), and 37°C (••••••••••••••••). It also shows the maximum cell growth obtained in the control at each condition of temperature (inset).
(0.53 MB TIF)

Figure S4 Growth curve of epimastigotes of *Trypanosoma cruzi* treated with T4C at 28°C and 5.5 pH: ▪ 0 mM, ▵ 0.1 mM, ○ 0.25 mM, ⧫ 0.5 mM, □ 0.75 mM, ▴1.0 mM. Inhibition control (•) was performed by incubation the parasites in the presence of 0.5 µM antimycin and 200 µM rotenone.
(0.55 MB TIF)

Figure S5 Growth curve of epimastigotes of *Trypanosoma cruzi* treated with T4C at 28°C and 6.5 pH: ▪ 0 mM, ▵ 0.1 mM, ○ 0.25 mM, ⧫ 0.5 mM, □ 0.75 mM, ▴1.0 mM. Inhibition control (─) was performed by incubation the parasites in the presence of 0.5 µM antimycin and 200 µM rotenone.
(0.45 MB TIF)

Figure S6 Sigmoidal dose-response curve of epimastigotes of *Trypanosoma cruzi*. Percentage of growth inhibition of *T. cruzi* epimastigotes at different temperatures and T4C. The curves represent the sigmoidal equation at pH 7.5 (─), 6.5 (---), and 5.5 (••••). It also shows the maximum cell growth obtained in the control at each condition of pH (inset).
(0.53 MB TIF)
