# Supplementary figures and images for: Actions of a Proline Analogue, L-Thiazolidine-4-Carboxylic Acid (T4C), on Trypanosoma cruzi
Source: PLoS One. 2009 Feb 20;4(2):e4534. doi: 10.1371/journal.pone.0004534 (PMC2645137; doi:10.1371/journal.pone.0004534)

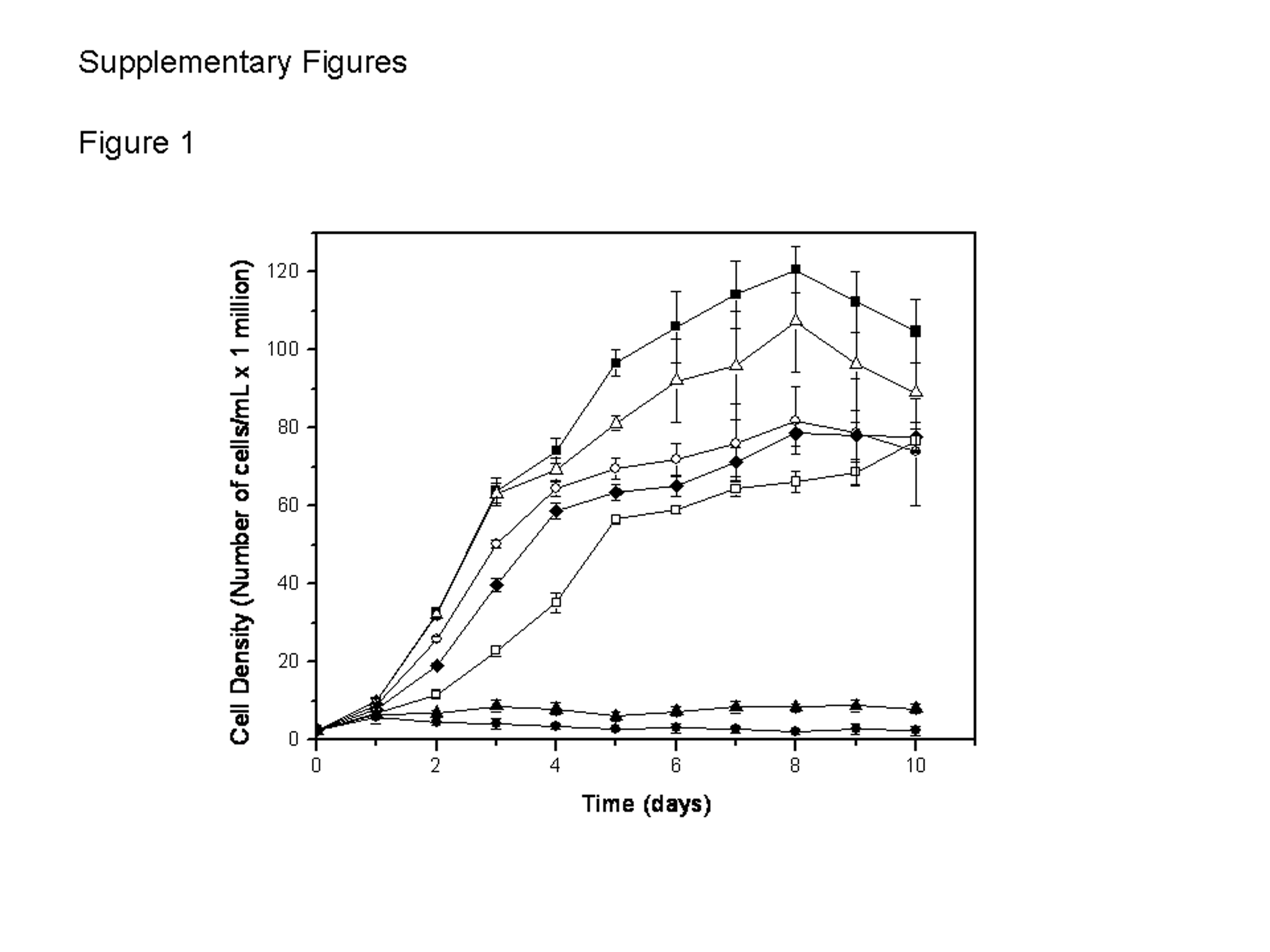

Supplement: Figure S1 — Growth curve of epimastigotes of Trypanosoma cruzi treated with T4C at 33°C and 7.5 pH: ▪ 0 mM, ▵ 0.1 mM, ○ 0.25 mM, ⧫ 0.5 mM, □ 0.75 mM, ▴1.0 mM. Inhibition control (─) was performed by incubation the parasites in the presence of 0.5 µM antimycin and 200 µM rotenone. (0.48 MB TIF) [file pone.0004534.s001.tif]

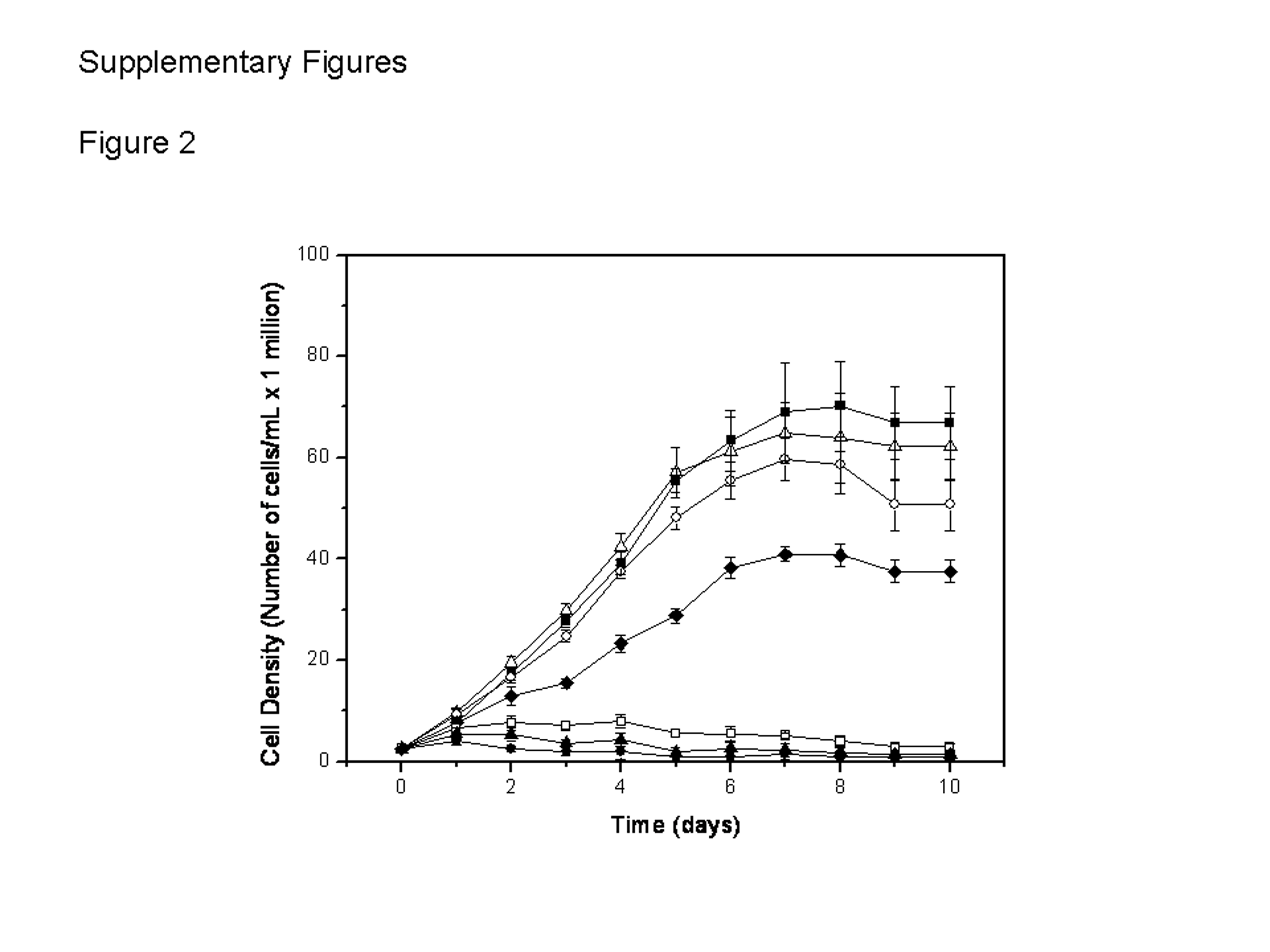

Supplement: Figure S2 — Growth curve of epimastigotes of Trypanosoma cruzi treated with T4C at 37°C and 7.5 pH: ▪ 0 mM, ▵ 0.1 mM, ○ 0.25 mM, ⧫ 0.5 mM, □ 0.75 mM, ▴ 1.0 mM. Inhibition control (─) was performed by incubation the parasites in the presence of 0.5 µM antimycin and 200 µM rotenone. (0.45 MB TIF) [file pone.0004534.s002.tif]

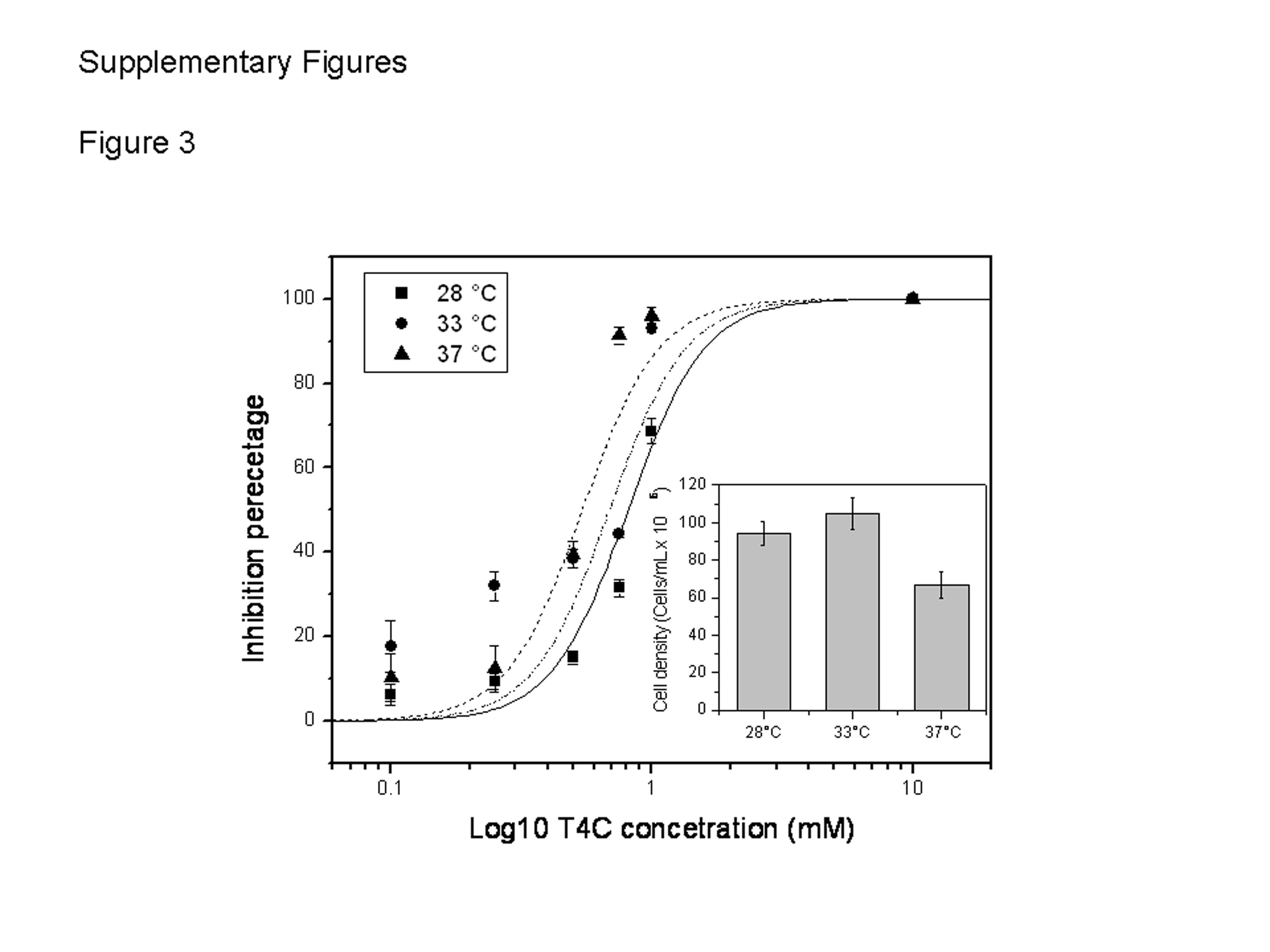

Supplement: Figure S3 — Sigmoidal dose-response curve of epimastigotes of Trypanosoma cruzi. Percentage of growth inhibition of T. cruzi epimastigotes at different temperatures and T4C. The curves represent the sigmoidal equation at 28°C (—), 33°C (---), and 37°C (••••••••••••••••). It also shows the maximum cell growth obtained in the control at each condition of temperature (inset). (0.53 MB TIF) [file pone.0004534.s003.tif]

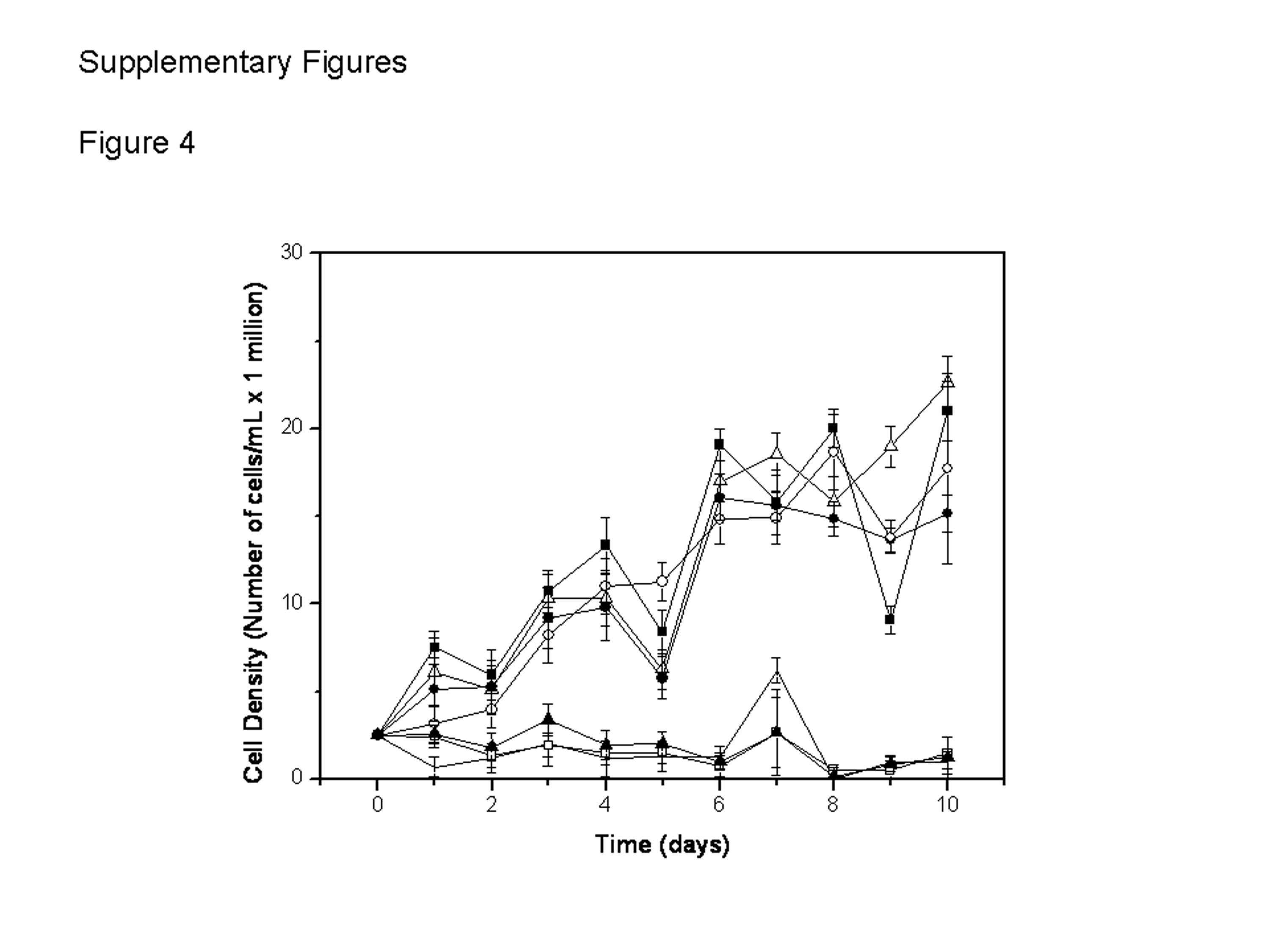

Supplement: Figure S4 — Growth curve of epimastigotes of Trypanosoma cruzi treated with T4C at 28°C and 5.5 pH: ▪ 0 mM, ▵ 0.1 mM, ○ 0.25 mM, ⧫ 0.5 mM, □ 0.75 mM, ▴1.0 mM. Inhibition control (•) was performed by incubation the parasites in the presence of 0.5 µM antimycin and 200 µM rotenone. (0.55 MB TIF) [file pone.0004534.s004.tif]

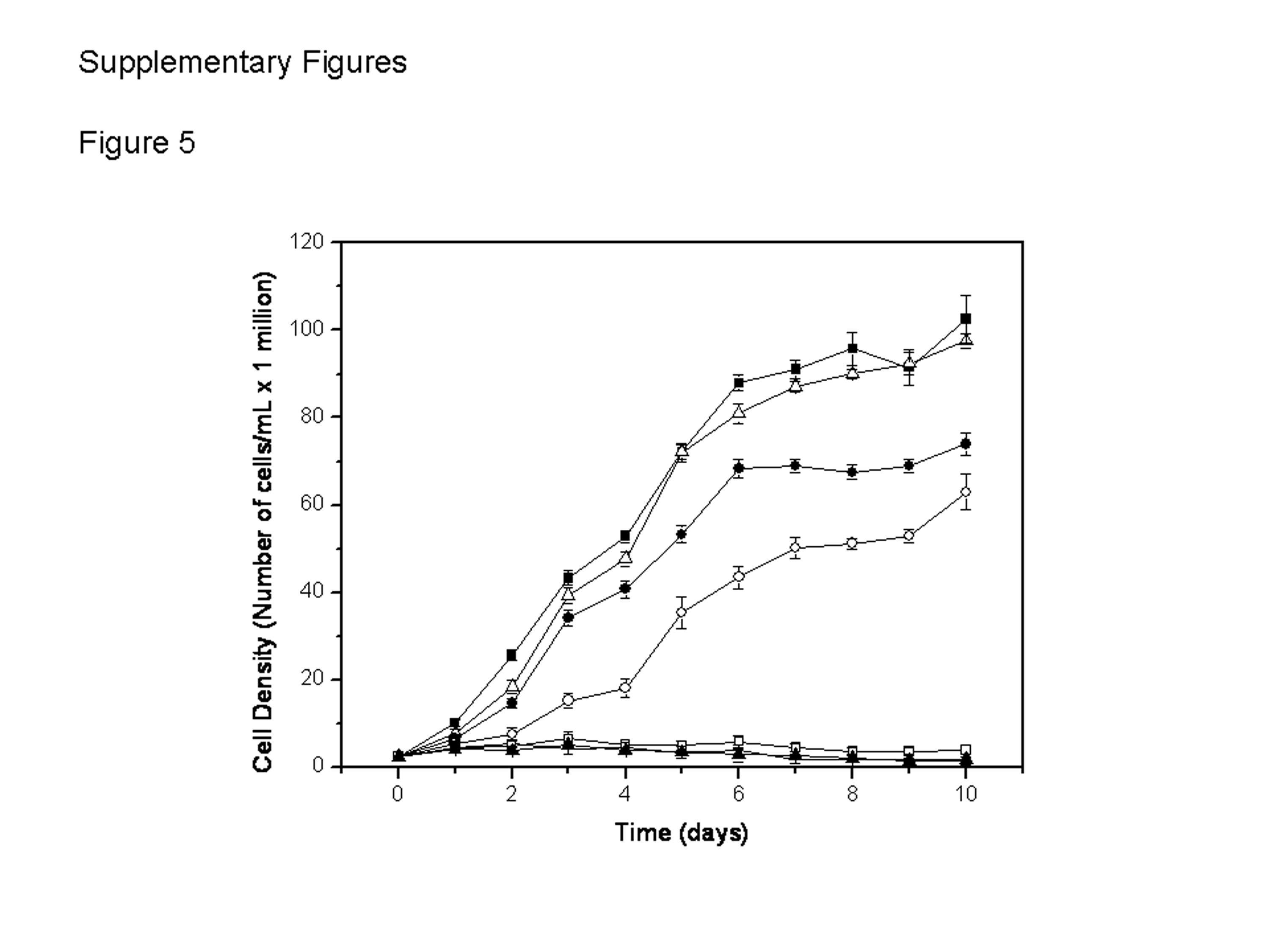

Supplement: Figure S5 — Growth curve of epimastigotes of Trypanosoma cruzi treated with T4C at 28°C and 6.5 pH: ▪ 0 mM, ▵ 0.1 mM, ○ 0.25 mM, ⧫ 0.5 mM, □ 0.75 mM, ▴1.0 mM. Inhibition control (─) was performed by incubation the parasites in the presence of 0.5 µM antimycin and 200 µM rotenone. (0.45 MB TIF) [file pone.0004534.s005.tif]

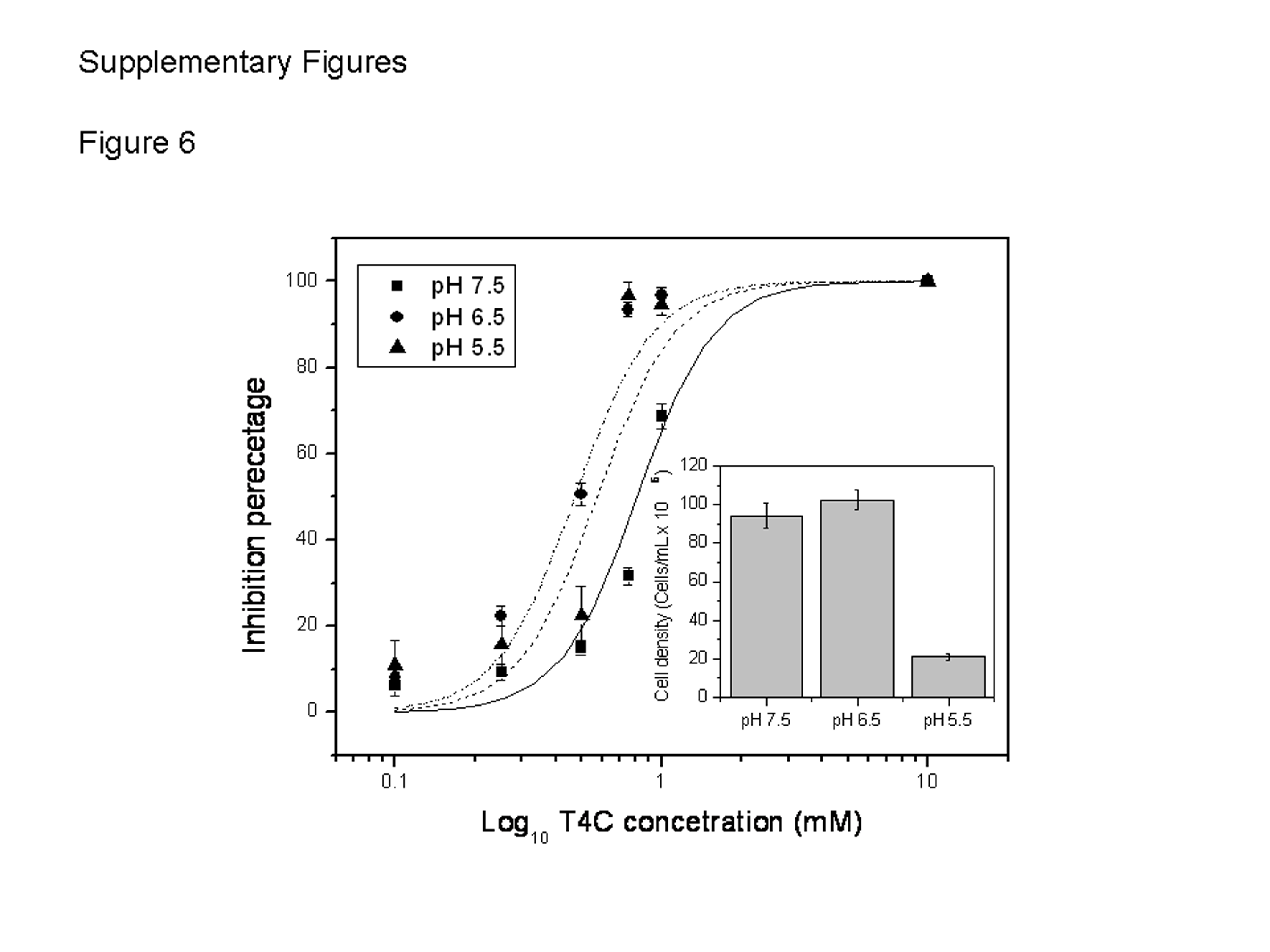

Supplement: Figure S6 — Sigmoidal dose-response curve of epimastigotes of Trypanosoma cruzi. Percentage of growth inhibition of T. cruzi epimastigotes at different temperatures and T4C. The curves represent the sigmoidal equation at pH 7.5 (─), 6.5 (---), and 5.5 (••••). It also shows the maximum cell growth obtained in the control at each condition of pH (inset). (0.53 MB TIF) [file pone.0004534.s006.tif]
